# Altered language–salience network connectivity in schizophrenia and differential associations with emotion regulation

**DOI:** 10.3389/fpsyt.2025.1695846

**Published:** 2025-12-18

**Authors:** Margherita Biondi, Marco Marino, Dante Mantini, Chiara Spironelli

**Affiliations:** 1 Padova Neuroscience Center, University of Padova, Padova, Italy; 2 Department of General Psychology, University of Padova, Padova, Italy; 3 Movement Control and Neuroplasticity Research Group, KU Leuven, Leuven, Belgium

**Keywords:** brain connectivity, emotional intelligence, fMRI, language network, msceit, salience network, schizophrenia

## Abstract

**Introduction:**

Emotion regulation is a key domain of social cognition, and its impairment contributes to poor psychosocial functioning in schizophrenia (SZ). The “Managing Emotions” (ME) branch of the Mayer-Salovey-Caruso Emotional Intelligence Test (MSCEIT) is widely used to assess this ability, yet its neural correlates remain unclear.

**Methods:**

We examined resting-state functional connectivity (rsFC) associated with MSCEIT-ME performance in 56 patients with schizophrenia and 56 healthy controls matched for age, sex, and years of education. Seed-based correlation analyses focused on three large-scale networks previously implicated in emotion regulation: the salience network (SN), the language network (LN), and the ventral attention network (VAN). Between-group differences and brain–behavior relationships were tested while controlling for IQ scores on the Wechsler Abbreviated Scale of Intelligence (WASI). False discovery rate Benjamini-Yekutieli (FDR-BY) correction was applied to all analyses.

**Results:**

Patients with SZ scored significantly lower on the MSCEIT-ME compared to healthy subjects (HCs). Moreover, SZ patients exhibited reduced left-lateralized rsFC between SN and LN regions relative to HCs. These findings indicate altered language–salience connectivity in schizophrenia and show that, while connectivity is associated with emotion regulation ability in healthy individuals, no significant brain–behavior association was detected in patients. Therefore, the neural mechanisms underlying emotion regulation deficits in schizophrenia remain to be clarified.

**Conclusion:**

Schizophrenia was characterized by altered left-lateralized language–salience connectivity. However, because no significant brain–behavior associations were found in patients, the neural basis of emotion-regulation deficits in schizophrenia remains unresolved, highlighting the need for network-level investigations in larger samples.

## 1 Introduction

Schizophrenia (SZ) is a severe psychiatric disorder that affects almost 24 million people globally (1). Its course is often characterized by poor long-term outcomes, including progressive deterioration, substantial functional disability, and persistent residual symptoms (2).

The complexity of SZ arises from the co-occurrence of heterogeneous symptoms involving perception, thought, cognitive processes, social functioning, and emotions (3). Among these, deficits in emotional intelligence have been increasingly recognized as a core feature, with a significant impact on patients’ psychosocial functioning (4, 5).

As first theorized by Mayer and Salovey (1990, 1997), emotional intelligence represents a domain of social intelligence that enables individuals to process emotional information and use it to guide thought and action. It consists of four interrelated abilities, assessed by the Mayer-Salovey-Caruso Emotional Intelligence Test (MSCEIT) (6): perceiving and identifying one’s own and others’ emotions; using emotions to enhance cognitive processes; understanding emotions and their transitions; and regulating emotions in oneself and others (7, 8).

Among these four, the Managing Emotions subtest (MSCEIT-ME) has received particular attention in SZ research. According to Mayer and Salovey (1997), the ability to regulate emotions is the most complex facet of emotional intelligence, as it requires higher-order reasoning to regulate emotions and select effective actions depending on context (8). Thanks to its structure (scenarios simulating real-life social situations), the MSCEIT-ME provides ecologically relevant information about emotion regulation, of which the domains assessed by the other MSCEIT subtests are considered prerequisites. Moreover, previous studies on SZ patients have shown that the MSCEIT-ME is consistently among the most impaired domains (5), shows good psychometric properties including reliability and test–retest stability (9, 10), and is more strongly associated with functional outcomes than the other MSCEIT subtests (11). Therefore, the MSCEIT-ME was selected by the National Institute of Mental Health (NIMH) as the measure of emotional components of social cognition in SZ in the MATRICS Consensus Cognitive Battery (MCCB) (11, 12). The MCCB is considered the gold-standard for the assessment of multi-domain cognitive functioning in SZ (13). Notably, DeTore et al. (2018) found that, among all the MCCB tests, the MSCEIT-ME uniquely predicted psychosocial functioning on the quality of life scale—including interpersonal and intrapsychic functioning—in a schizophrenia-schizoaffective disorder group, even after controlling for other cognitive functions and symptoms (12). The widespread use of MCCB in both research and clinical settings facilitates comparability across studies and supports standardized, clinically relevant assessment of emotion regulation in SZ. Even in the open-access dataset used in the present study, patients were administered the MCCB and, therefore, the MSCEIT-ME for the assessment of emotion regulation.

Although deficits in emotion regulation are well documented in SZ (14–17), the underlying neural processes remain poorly understood. Evidence so far has linked poor MSCEIT-ME performance in patients to reduced grey matter density in the left Parahippocampus Gyrus and the right Posterior Cingulate (18), increased spontaneous activity in the right Middle Frontal Gyrus (19), and decreased task-evoked activity in the right Temporo–Parietal Junction (20).

Given the complexity of the ability to regulate emotions (8), Killgore and colleagues (2017) suggested that such multifaceted ability may be better captured by resting-state functional connectivity (rsFC) patterns (21). Resting-state networks (RSNs) are sets of brain regions that exhibit intrinsic functional coupling in the absence of task demands. Studying their connectivity provides insight into how brain architecture supports performance (22, 23) and how it is altered in disorders such as SZ (24). Clinically, rsFC offers the advantage of not requiring active task engagement during fMRI scanning, which is particularly suitable for patients who may struggle with cooperation or task performance (25).

Recent studies have begun to examine the relationship between rsFC and MSCEIT-ME in SZ, implicating regions such as the left superior parietal lobule (26, 27), insular subregions (28), right precuneus (29), and the cerebellum (27, 30). However, RSN-level analyses remain limited. To date, only Jimenez et al. (2019) investigated the contribution of multiple RSNs (visual, sensorimotor, auditory, default mode, executive control, frontoparietal, and cerebellar), but found no significant associations with MSCEIT-ME (31). Critically, other RSNs with known relevance to SZ and emotion regulation have yet to be examined (32).

In this study, we aimed to fill this gap by investigating specific resting-state connectivity patterns and the correlation with the ability to regulate emotions in SZ patients compared to healthy controls (HCs) across three RSNs relevant to emotion regulation and unexplored in association with MSCEIT-ME in a SZ sample: the Salience Network (SN), the Language Network (LN), and the Ventral Attention Network (VAN). The SN is central to emotion regulation and social cognition, as it plays a pivotal role in detecting and integrating emotionally salient stimuli to guide adaptive responses (33). Abnormal SN functioning has been consistently linked to impairments in salience attribution and social processing in SZ (34). The LN encompasses linguistic areas consistently altered in SZ, and key symptoms like auditory verbal hallucinations, thought and speech disorders, have been suggested to stem from underlying neural dysfunctions in language-related brain regions (35). Also, the LN is involved in emotional processing (36): for example, it has been shown that it contributes to inner speech, the form of internal language serving a range of cognitive functions, including emotion regulation (37). Finally, the VAN, the spatial right-hemisphere homolog of the LN, is implicated in the orientation of attention towards emotional cues (38, 39), and dysfunction within this network could therefore contribute to deficits in attention shifting and emotion monitoring, both of which are central to emotion regulation ability.

## 2 Materials and methods

### 2.1 Participants

fMRI data used in this study were downloaded from COINS (https://coins.trendscenter.org), an online, open-access database comprising various datasets designed for neuroimaging research. We outlined the following criteria in a query on COINS: 1) availability of resting-state fMRI data; 2) availability of T1 structural MRI (sMRI) data; 3) availability of the MSCEIT-ME scores for both SZ patients and HCs (6). COINS returned the COBRE project as the suitable dataset according to our query. Thus, the dataset used in this study was entirely obtained from the COBRE project, which gathers neuroimaging and clinical data from individuals recruited at the University of New Mexico (UNM) Psychiatric Center, the Raymond G. Murphy Veterans Affairs Medical Center, and other clinics in the Albuquerque metropolitan area (USA). Neuroimaging data were all acquired using the same scan and the same acquisition protocol (see Paragraph 2.3). SZ patients were included based on the following criteria: 1) a diagnosis of SZ confirmed by two independent psychiatrists using the DSM-IV Structured Clinical Interview for Axis I Disorders (SCID) (40); 2) evidence of clinical stability for at least 3 months before and during MRI scanning; 3) age between 18 and 65 years. HCs were also recruited in Albuquerque, with the following inclusion criteria based on the SCID-Non-Patient edition: 1) no diagnosis of any current or past Axis I psychiatric disorder; 2) no history of head trauma with loss of consciousness greater than 5 minutes; 3) no substance abuse, addiction, or antidepressant use in the past 5 months; 4) absence of psychotic disorders among first-degree relatives. Demographic data for both groups (age, sex distribution, years of education) and clinical data about the patients (age of onset, illness duration, medication) were collected. All participants involved in the COBRE project provided written informed consent. COBRE-funded data collection took place at the Mind Research Network, subject to the licensing procedure 5P20RR021938/P20GM103472 from the National Institutes of Health (NIH) to Dr. Vince Calhoun. All data were anonymized before access to protect participants’ privacy. sMRI and fMRI data were visually inspected to ensure the suitability of the sample.

### 2.2 Assessment

The MSCEIT-ME (i.e., the “managing emotions” subtest of the MSCEIT [6]) was used to assess the ability to regulate emotions in SZ patients and HCs. It consists of two tasks: the emotion management task and the social management task. Both tasks present brief vignettes describing social situations, each followed by a set of possible actions. In the emotion management task, participants were asked to evaluate how each action would affect the emotional state or behavior of the individual or others involved in the scenario. In the social management task, participants had to rate each action based on its social effectiveness. In both cases, responses were provided using a 5-point Likert scale. Raw scores are calculated by assigning a numerical value to each individual’s response based on how closely it matches the response given by the general population. In this way, a performance score is obtained, that is representative of the ability to regulate one’s own and others’ emotions in an adaptive manner, which is considered a key component of emotional intelligence and social cognition. Differences between SZ patients and HCs on MSCEIT-ME scores were assessed using a univariate ANOVA. In addition, as emotion regulation requires working memory, attention, and executive function – all functions typically impaired in schizophrenia – we decided to control for general cognitive ability. The COBRE dataset also included the Wechsler Abbreviated Scale of Intelligence (WASI; [41]), therefore we included the WASI total scores, as well as the Verbal WASI (WASI-V) and the performance WASI (WASI-P) subscales and general indices of participants’ cognition. Differences between SZ patients and HCs on all WASI scores were assessed using separate univariate ANOVAs.

### 2.3 MRI data acquisition

MRI data were acquired using a 3T Siemens MR scanner (Trio, Siemens Healthcare, Erlangen, Germany). The acquisition protocol allowed for sagittal-gradient echo-scout images through the midline to obtain image slices that were axial, oblique, and parallel to the antero-posterior commissure (AC-PC) line; in particular, oblique slices were used to minimize the orbitofrontal susceptibility artifact. Resting-state fMRI data were collected over 5 minutes using a single-shot, gradient echo-planar pulse sequence with lipid suppression, with the following parameters: number of volumes = 150, TR = 2000 ms, TE = 29 ms, flip angle = 75°, FOV = 240 mm, matrix size = 64 × 64, 33 slices, voxel size = 3.75 × 3.75 × 4.55 mm³. High-resolution T1-weighted sMRI images were collected using a multi-echo MP-RAGE sequence (5 echoes) with parameters: TE (Echo Times) = 1.64, 3.5, 5.36, 7.22, 9.08 ms, TR (Repetition Time) = 2.53 s, TI (Inversion Time) = 1.2 s, flip angle = 7°, Number Of Excitations (NEX) = 1, slice thickness = 1 mm, FOV (Field Of View) = 256 mm, resolution = 256 × 256. The first image from each acquisition was removed to account for T1 equilibrium effects. Detailed scanning parameters are available at the COINS website.

### 2.4 MRI data preprocessing

**Figure 1:**
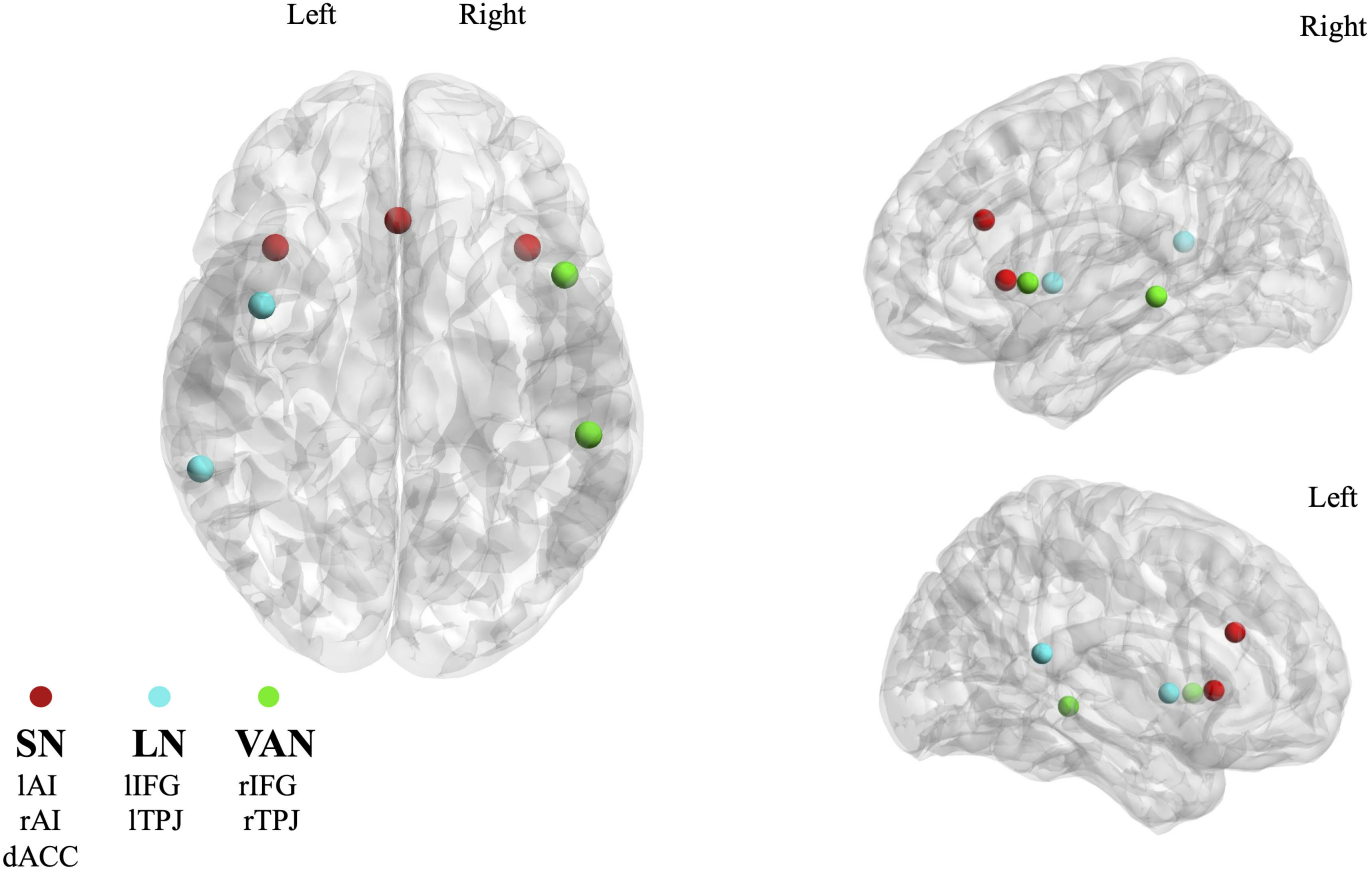
Anatomical positions of the 7 ROIs used in the study, subdivided into the corresponding 3 RSNs: Salience Network (SN, red), Language Network (LN, light blue), Ventral Attention Network (VAN, green). MNI coordinates of the ROIs can be found in **Table 1**, whereas the full names are listed here: left Anterior Insula (lAI), right Anterior Insula (rAI), dorsal Anterior Cingulate Cortex (dACC), left Inferior Frontal Gyrus (lIFG), left Temporo-Parietal Junction (lTPJ), right Inferior Frontal Gyrus (rIFG), right Temporo-Parietal Junction (rTPJ).

Resting-state fMRI data were preprocessed through an automated pipeline developed using the Statistical Parametric Mapping 12 (SPM12) software (https://www.fil.ion.ucl.ac.uk/spm/software/spm12/), including motion correction, spatial alignment to the T1 structural image, band-pass filtering (0.01–0.1 Hz), white matter, cerebrospinal fluid and global signals regression, and spatial smoothing at 6 mm full width half maximum (FWHM) (42). To control for head motion, we calculated the framewise displacement (FD), computed as the sum of the absolute values of the derivatives of the translational and rotational realignment estimates at every timepoint (43). Outlier detection and scrubbing were performed with an FD threshold of 0.5 mm (43). The quality control of MRI data and the empirical evaluation of residual motion contribution in the functional connectivity estimations are reported in **Section 1 of the Supplementary Materials** (**Supplementary Table S1**). The regions of interest (ROIs) of the RSNs we investigated were defined based on previous studies: the SN included the left Anterior Insula (lAI), the right Anterior Insula (rAI), and the dorsal Anterior Cingulate Cortex (dACC) according to Mantini et al. (2013) (44); the LN included the left Inferior Frontal Gyrus (lIFG) and the left Temporo-Parietal Junction (lTPJ) and the VAN included the right Inferior Frontal Gyrus (rIFG) and the right Temporo-Parietal Junction (rTPJ) according to Samogin et al. (2020) (45). The Montreal Neurological Institute (MNI) coordinates of the ROIs of each network (reported in **Table 1**) were projected into individual MRI space by inverting the spatial transformation required to register the individual MR image to MNI space, and a spherical ROI with a 6 mm radius was defined around each set of coordinates (**Figure 1**). The time-series across all the voxels within each spherical ROI were averaged and considered as representative of the activity in the whole ROI.

**Table 1:** List of ROIs, with their respective MNI spatial coordinates, of the RSNs of interest.

RSN	ROI	MNI coordinates
Salience Network (SN)	lAI (left Anterior Insula) rAI (right Anterior Insula) dACC (dorsal Anterior Cingulate Cortex)	[-36,22,2] [38,22,2] [2,30,24]
Language Network (LN)	lIFG (left Inferior Frontal Gyrus) lTPJ (left Temporo-Parietal Junction)	[-47,14,1] [-54,-33,-4]
Ventral Attention Network (VAN)	rIFG (right Inferior Frontal Gyrus) rTPJ (right Temporo-Parietal Junction)	[42,5,1] [60,-43,16]

### 2.5 RSN connectivity analysis

We analyzed the connectivity patterns between pairs of ROIs by computing the Pearson correlation of their activity, resulting in a 7×7 connectivity matrix. Within-network connectivity was defined as the average correlation between pairs of ROIs within the same network, while between-network connectivity was defined as the average correlation between all the possible pairs of ROIs belonging to two different networks (46). This analysis was conducted for both the SZ and HC groups. Next, group differences in connectivity were assessed using an unpaired two-sample t-test on connectivity values between each pair of ROIs. To control for general cognitive ability, WASI total IQ scores were included as a covariate. To correct for multiple comparisons, the False Discovery Rate Benjamini-Yekutieli (FDR-BY) method was applied, with a significance threshold set at *q* < 0.05 (47).

### 2.6 Correlation between RSN connectivity values and MSCEIT-ME scores

We computed the Spearman’s correlation coefficients between the average rsFC values of each pair of ROIs and the MSCEIT-ME scores, separately for SZ and HC groups, controlling for WASI total IQ scores. The significance level of the correlations was set to *q* < 0.05 (FDR-BY corrected). To evaluate the dependence of brain–behavior associations on preprocessing choices, we additionally repeated the brain–behavior analyses on data processed identically, but without global signal regression (GSR). Furthermore, complementary analyses on the association between rsFC values and WASI scales are reported in **Section 2 of the Supplementary Materials** (**Supplementary Figures S1**-**Supplementary Figures S3**).

## 3 Results

### 3.1 Demographic and clinical data

Demographic and clinical characteristics are reported in **Table 2**. The final sample included 56 SZ patients and 56 HCs, matched for age, sex, and years of education. For SZ patients, **Table 2** also summarizes average age of onset, illness duration, and antipsychotic treatment (converted to olanzapine and chlorpromazine equivalents). Since the medication doses of our clinical sample show large variability, we also checked for the impact of olanzapine-equivalent and chlorpromazine-equivalent doses on both patients’ behavioral data and patients’ functional connectivity. Results are reported in **Section 3 of the Supplementary Materials**. Notably, behavioral analysis revealed no significant correlations between drugs and patients’ behavioral performance on MSCEIT-ME scores or on WASI scales.

**Table 2:** Demographic and clinical characteristics of the studied sample.

Demographic and clinical characteristics	SZ (n=56)	HC (n=56)	Statistics
Age	37.34 ± 12.7	38.16 ± 13	*t*_123_ = -0.34, *n.s.*
Sex (M/F)	45/11	41/15	*χ*^2^ = 0.8, *n.s.*
Years of education	12.95 ± 1.55	13.45 ± 1.5	*t_123_ =* -1.73, *n.s.*
Age of onset	21.68 ± 7.45	–	–
Illness duration (years)	16.16 ± 12.26	–	–
Total olanzapine equivalent dose (mg)	15.38 ± 10.74	–	–
Total chlorpromazine equivalent dose (mg)	387.11 ± 321.82	–	–

Mean ± standard deviation (SD) of age, years of education, age of onset, illness duration, and pharmacological treatment were considered.

### 3.2 MSCEIT-ME performance

SZ patients and HCs obtained significantly different scores on MSCEIT-ME, with patients scoring lower (M = 90.2, SD = ± 10.58) than controls (M = 98.05, SD = ± 9.5). A univariate ANOVA revealed a significant main effect of group on scores (*F*_1,110_ = 17.07, *p* < 0.001). In addition, the WASI analyses on both total IQ scores and verbal/performance IQ scores were significant too (*F*_1,110_ = 10.83, *p* = 0.001, *F*_1,110_ = 5.83, *p* = 0.017, and *F*_1,110_ = 11.04, *p* = 0.001, respectively), revealing that, compared with healthy controls, the general cognitive ability, the verbal and the performance skills were all reduced in SZ patients (HC group: WASI total IQ scores M = 110.02 ± 11.94, Verbal IQ M = 106.32 ± 11.75; Performance IQ = 111.46 ± 12.90; SZ group = WASI total IQ scores M = 100.98 ± 16.73, Verbal IQ M = 99.89 ± 16.10; Performance IQ = 102.00 ± 16.96).

However, the correlations between the MSCEIT-ME scores and every WASI score were not significant (HC group: all *r*s < 0.029, all *p*s n.s.; SZ group = all *r*s < 0.039, all *p*s n.s.), suggesting that there were no significant associations among the scores obtained to emotion regulation and participants’ cognitive functioning. In any case, to control for possible confounding effects depending on cognitive functioning, the WASI total IQ scores were included in all the connectivity analyses carried out withing and between groups.

### 3.3 RSN connectivity patterns

**Figure 2:**
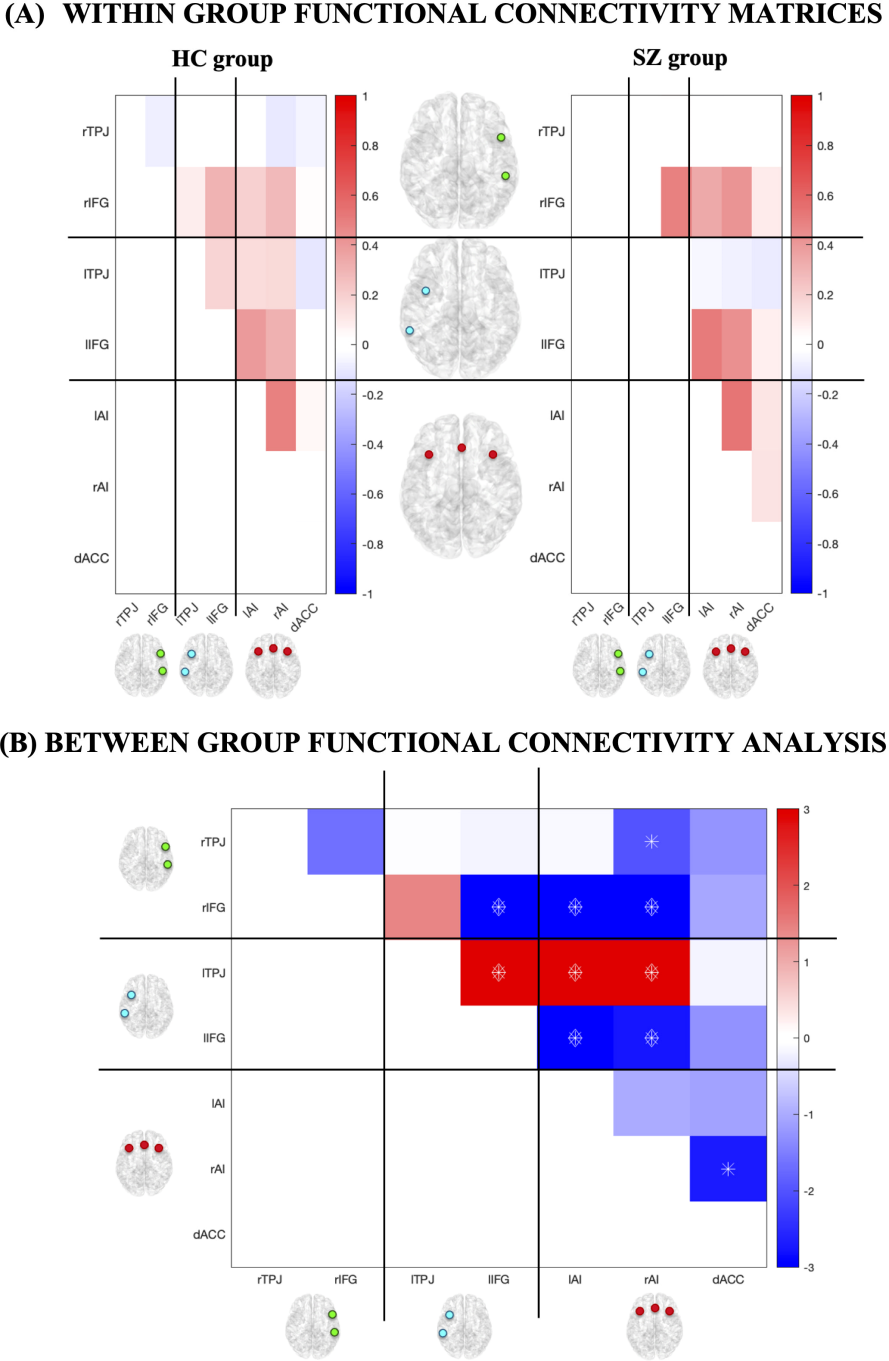
Functional connectivity values and their differences between all possible pairs of ROIs for SZ and HC groups. **(A)** The matrices represent the average connectivity values between all possible pairs of ROIs, separate for HC and SZ groups. **(B)** For each ROI pair, significant differences between HC and SZ groups at *p* < 0.05 are marked with an asterisk, those at *q* < 0.05 (FDR-BY corrected) with a diamond.

Group differences in rsFC, controlling for WASI total IQ scores, are shown in **Figure 2**. Compared to patients, HCs exhibited higher rsFC within the LN (lTPJ–lIFG pair, Cohen’s *d* = 0.66) and between the LN and SN (lTPJ–lAI and lTPJ–rAI pairs, Cohen’s *d*s = 0.78 and 0.86, respectively) (FDR-BY corrected) (**Figure 2B**). Conversely, SZ patients showed higher rsFC between the VAN and LN (rIFG–lIFG pair, Cohen’s *d* = -0.76), between the VAN and SN (rIFG–lAI and rIFG–rAI pairs, Cohen’s *d*s = -0.58 and -0.60, respectively), and between the LN and SN (lIFG–lAI and lIFG–rAI pairs, Cohen’s *d*s = -0.60 and -0.53, respectively) (FDR-BY corrected) (**Figure 2B**).

### 3.4 Correlation between RSN connectivity values and MSCEIT-ME scores

**Figure 3:**
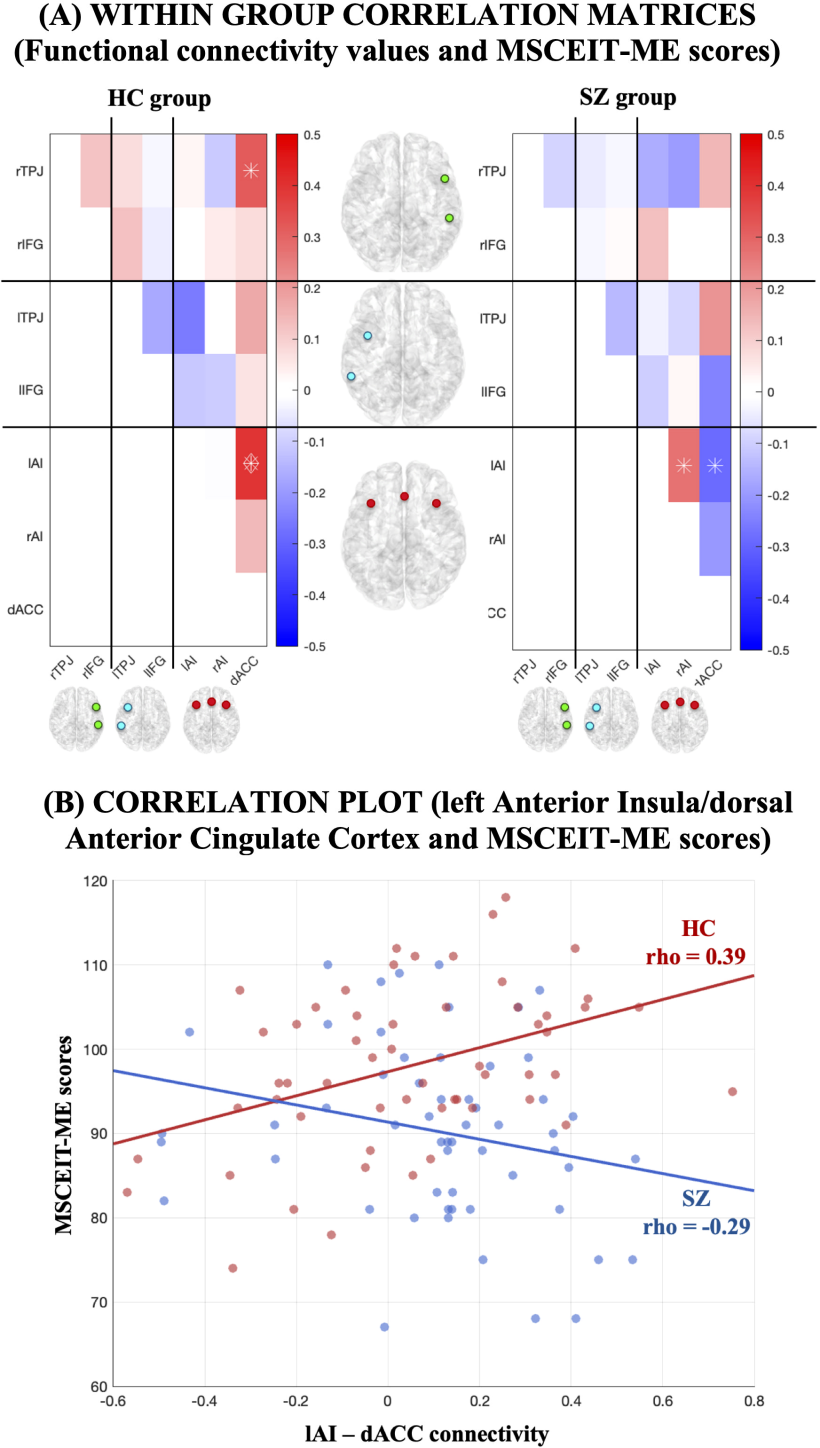
**(A)** Correlation between MSCEIT-ME scores and the connectivity values between all possible pairs of ROI for SZ and HC groups. Correlation with *p* < 0.05 is marked with an asterisk, those at *q* < 0.05 (FDR-BY corrected) with a diamond. **(B)** Correlation plot showing the correlations (FDR-corrected) for HC (red color) and (no FDR-corrected) for SZ (blue color) between left Anterior Insula (lAI) and dorsal Anterior Cingulate Cortex (dACC). WASI total scores were included as covariate.

After controlling for WASI total IQ scores, we found significant correlations between MSCEIT-ME scores and some ROI pairs (**Figure 3A**). In the HC group, higher MSCEIT-ME scores were associated with higher rsFC values within the SN (lAI-dACC pair) (*rho* = 0.39, *p* = 0.0028). This result survived the FDR-BY correction (*q* = 0.0283; **Figure 3B**). In the SZ group, lower MSCEIT-ME scores were associated with higher rsFC values within the SN (lAI-dACC pair) (*rho* = -0.29, *p* = 0.0342); however, this result did not survive the FDR-BY correction (**Figure 3B**). Notably, all connectivity–behavior associations in this study were present only when global signal regression was included in preprocessing; without this processing step, no correlations reached statistical significance.

Finally, as past research has consistently demonstrated that emotion regulation involves the interplay between executive control/frontoparietal networks and default mode network regions, we carried out a complementary analysis focused on these rs-fMRI networks (**Supplementary Figures S5** and **Supplementary Table S2** in the Supplementary Materials). Results are reported in **Section 4 of the Supplementary Materials** (**Supplementary Figures S6**, **Supplementary Figures S7**).

## 4 Discussion

### 4.1 Key findings

In the present study, we investigated the rsFC patterns across three RSNs relevant to emotion regulation, the Salience, Language, and Ventral Attention Networks, and their association with a well-validated measure of emotion regulation, the MSCEIT-ME. To the best of our knowledge, this is the first time these RSNs have been studied in association with the MSCEIT-ME, the subtest of the emotional intelligence test by Mayer, Salovey, and Caruso (2003) (6), that was selected by the NIMH to study the emotion regulation ability as a key aspect of social cognition in SZ (11, 12).

Our findings first corroborate the robust evidence that patients with SZ exhibit significant deficits in emotion regulation. Indeed, our SZ sample scored significantly lower on the MSCEIT-ME compared to healthy subjects, replicating previous work (14–17). This result is consistent with the notion that regulating emotions represents the most complex facet of emotional intelligence, requiring higher-order reasoning to regulate emotions and select adaptive responses depending on context (6, 8).

By confirming the magnitude of MSCEIT-ME impairment in SZ, our study also reinforces the importance of this construct for understanding broader psychosocial dysfunction. Emotion regulation deficits are closely tied to reduced social functioning and impaired quality of life, and have been shown to predict long-term functional outcomes in SZ beyond symptom severity alone (48, 49). Thus, investigating the neural substrates of this ability is not only theoretically relevant, but also clinically meaningful.

### 4.2 Network-level findings

Concerning the RSNs, we found that SZ patients and HCs were characterized by different rsFC patterns, involving brain regions with a key role in language and social functions.

First, we found that HCs were characterized by higher rsFC within the LN (lTPJ–lIFG pair) and between the LN and SN (lTPJ–lAI and lTPJ–rAI pairs) compared to SZ patients. Thereby, the lTPJ – the posterior region of the LN engaged in language processing as well as social cognition (50) – was less connected with the lIFG and the bilateral AI in our SZ sample. Notably, previous studies showed that altered lTPJ–IFG connectivity might be involved in auditory verbal hallucinations (51, 52), and neuromodulation approaches such as rTMS and tDCS targeting this region have shown clinical benefits on hallucinations, accompanied by altered functional coupling with IFG and insula (53, 54).

Our finding of altered connectivity between LN and SN (lTPJ–lAI and lTPJ–rAI pairs) in SZ patients points to a broader breakdown in the integration of socio-linguistic and salience processing. The AI, a key hub of the SN, supports saliency detection, attention switching, emotional responses, empathy, and social cognition (55). Converging evidence from structural MRI and large-scale meta-analyses highlights the insula as one of the most consistently affected cortical regions in schizophrenia, showing reductions in volume and thickness (56, 57), abnormal task-related activity (58), and altered rsFC patterns (59), particularly in patients with auditory verbal hallucinations (60). Taken together, these findings identify the lTPJ and AI as critical hubs whose diminished connectivity may represent a core neurobiological alteration in schizophrenia, implicated in psychotic symptoms and potentially relevant to emotion-regulation processes more broadly.

Along with diminished left-lateralized rsFC between the LN and SN, SZ patients also exhibited increased rsFC both frontally and bilaterally compared to HCs. Indeed, we found that SZ patients showed higher rsFC between the VAN and LN (rIFG–lIFG pair), between the VAN and SN (rIFG–lAI and rIFG–rAI pairs), and between the LN and SN (lIFG–lAI and lIFG–rAI pairs). It is important to highlight that, while the rsFC within the LN on the left hemisphere (lTPJ-lIFG pair) was reduced in our SZ sample, a bilateral increase in rsFC between the lIFG and its right homologous emerged. The rIFG is a key component of the VAN, a RSN with distinctive features: primarily implicated in attentive processes driven by salient stimuli (61) – including those with emotional content (39) – it is also the symmetrical LN homologous in the right hemisphere (38). Chang et al. (2015) also found an increased inter-IFG coupling in SZ patients with auditory verbal hallucinations, suggesting how diminished asymmetry in the IFG might be related to inter-hemispheric rsFC (62), and this might have a pathophysiological role for symptoms related to linguistic processes like auditory verbal hallucinations (63). Nevertheless, this bilateral pattern aligns with evidence for atypical interhemispheric organization in SZ, that is, a reduced functional lateralization/asymmetry (64), which in our case extended also to the SN (rIFG-lAI, rIFG-rAI, lIFG-lAl, lIFG-rAl pairs). Notably, Zhang et al. (2022), who studied rsFC of the insula subregions in association with MSCEIT-ME in patients with first-episode SZ, also found increased rsFC between the rIFG and lAI in patients compared to HCs (28). According to the authors, the increased activation of the IFG might trigger excessive and inappropriate emotional responses from the AI, potentially contributing to emotional dysregulation (28).

Regarding brain–behavior associations, only healthy controls showed a significant positive correlation between MSCEIT-ME scores and rsFC within the salience network (lAI–dACC). No connectivity–behavior associations survived correction in the SZ group, indicating that the current dataset does not provide statistically reliable evidence that rsFC is linked to emotion regulation performance in schizophrenia.

Although an uncorrected negative trend in SZ paralleled prior findings (e.g., 28), this trend should be considered exploratory and hypothesis-generating rather than interpretively meaningful. Larger samples and confirmatory studies are required before drawing conclusions about the neural correlates of emotion-regulation deficits in SZ. Bajaj and Killgore (2021) found that the effective connectivity strength between the connection from the dACC to the left insula was positively associated with the scores on the understanding emotions MSCEIT subtest (65). Notably, dACC appears to have a key role in sustaining good performance in healthy individuals, particularly in light of its involvement in emotional processes such as emotion appraisal and regulation (66). In our SZ sample, the association between lAI-dACC connectivity values and MSCEIT-ME scores goes in the opposite direction to HCs (i.e., higher lAI-dACC connectivity values correlated with lower MSCEIT-ME scores in SZ sample), but this effect did not survive the FDR-BY correction. However, it should be noted that, in a previous study, Zhang et al. (2022) found that patients with first-episode SZ exhibited increased rsFC between the AI and the anterior middle cingulate cortex (located just posterior to the dACC), and their increased rsFC correlated negatively with MSCEIT-ME scores (28). The authors suggested that this mechanism might explain the pathologically enhanced detection of emotional salience stimuli in SZ (28). Taken together, while the correlation in our SZ sample should be interpreted with caution, its alignment with previous evidence raises the possibility of a link between altered salience network connectivity and deficits in emotion regulation measured with MSCEIT-ME in SZ, which warrants further investigation with larger samples.

### 4.3 Clinical implications

Our findings have clinical implications. In SZ, emotion regulation ability has been shown to correlate positively with objective quality-of-life indicators such as occupational engagement and social contacts (48), and to predict social and occupational outcomes longitudinally (49). This highlights the importance of including emotion regulation assessment as a core element of functional evaluation, complementing traditional symptom-based measures. Targeting this ability through psychosocial interventions – such as social cognition training or emotion regulation therapies – may therefore yield meaningful improvements in daily functioning.

Moreover, evidence from related clinical populations supports the potential of neuromodulation approaches. For example, a study comparing healthy individuals with adults affected by generalized social phobia and generalized anxiety disorder– two conditions marked by emotional dysregulation – found that HCs recruited the dACC more strongly during emotion regulation, whereas generalized social phobia and generalized anxiety disorder patients did not (67). This suggests that the dACC plays a critical role in adaptive regulation. Accordingly, stimulation techniques targeting the dACC may enhance emotion regulation capacities, possibly offering a therapeutic avenue for SZ and other disorders characterized by emotional dysregulation.

Regarding the use of the resting-state approach, it is clinically relevant that this method avoids the confounding effects of task-based constraints; therefore, it eases the burden on the experimental design and the participants’ compliance, enabling the investigation of brain functional organization also in patients who are poorly cooperative or who struggle with performing specific tasks, such as SZ patients.

Together, these clinical perspectives underscore the translational potential of linking MSCEIT-ME deficits to specific neural connectivity profiles, while also highlighting the need for further studies that address the methodological and sample-related limitations of the present work.

## 5 Limitations

The present study has some limitations that should be acknowledged. First, we focused on the MSCEIT-ME subtest, as it was the only one available in the COBRE database, reflecting its selection by the NIMH for inclusion in the MATRICS Consensus Cognitive Battery. Future studies may benefit from employing the full MSCEIT to enable a more comprehensive assessment of distinct emotional intelligence domains and their neural correlates. Second, resting-state fMRI data were collected over 5 minutes in the COBRE dataset. Although this duration meets the minimum standard for group-level connectivity analyses, it is relatively limited for capturing individual variability in brain-behavior associations (68). Extending resting-state scan durations in future datasets would increase sensitivity to individual differences. Third, although our sample size was in line with previous neuroimaging work, it remains relatively modest, limiting generalizability. Replication in larger and more diverse cohorts will be essential to confirm the robustness of our findings. Fourth, all patients were receiving antipsychotic medication, which may have influenced resting-state connectivity patterns, particularly within salience and language networks. While this reflects typical clinical conditions, studies on unmedicated or first-episode patients are needed to disentangle medication effects from disease-related alterations. Fifth, the cross-sectional design prevents causal inferences; longitudinal research is necessary to determine whether altered connectivity drives deficits in emotion regulation or represents a downstream effect of broader pathophysiology. Moreover, longitudinal investigations are essential to relate MSCEIT-ME scores with functional outcomes or treatment targets. Sixth, our use of a seed-based connectivity approach focused on predefined networks, which provides specificity but has some limitations: it is sensitive to seed placement, it is limited to an arbitrary selection of regions of interest, and it may overlook broader whole-brain or graph-theoretical alterations. In future studies, the inclusion of complementary validation analyses using alternative connectivity approaches (e.g., dynamic connectivity, graph metrics) could yield additional insights into the network-level mechanisms underlying socio-emotional dysfunction in schizophrenia. Finally, all brain–behavior associations identified in this study were dependent on the use of global signal regression (GSR) during preprocessing. When GSR was omitted, no connectivity–behavior correlations reached significance. Because GSR may reduce widespread confounds but can also alter resting-state correlation structure and introduce anticorrelations (69–71), these findings should be interpreted cautiously. The present results therefore reflect the specific preprocessing pipeline used here rather than demonstrating that GSR reveals underlying ‘true’ associations. Future work should evaluate the robustness of brain–behavior relationships across multiple preprocessing strategies, including pipelines with and without GSR.

## 6 Conclusion

The present work provides novel insights into the relationship between emotion regulation ability and resting-state functional connectivity within three networks relevant to emotion regulation but largely unexplored in this context (SN, LN, and VAN). Patients with schizophrenia showed impaired MSCEIT-ME performance and altered left-lateralized rsFC between language and salience network regions, including TPJ, IFG, AI, and dACC – areas implicated in emotion-regulation processes in prior literature. In the present study, however, we did not observe significant brain–behavior associations in schizophrenia, whereas healthy individuals displayed connectivity patterns more consistent with effective emotion regulation.

MSCEIT-ME performance was associated with salience-network connectivity only in healthy individuals. No significant connectivity–behavior associations were detected in schizophrenia after correction for multiple comparisons. Therefore, although emotion regulation remains a clinically important domain, the present data do not allow conclusions about its neural underpinnings in schizophrenia. Future research with larger samples and advanced preprocessing pipelines will be needed to determine whether specific connectivity patterns relate to emotion-regulation deficits in schizophrenia and whether such patterns could eventually inform therapeutic strategies.

## Data Availability

Publicly available datasets were analyzed in this study. This data can be found here: COINS (https://coins.trendscenter.org).
