# Supplementary material for: Altered language–salience network connectivity in schizophrenia and differential associations with emotion regulation
Source: Front Psychiatry. 2025 Dec 18;16:1695846. doi: 10.3389/fpsyt.2025.1695846 (PMC12756178; doi:10.3389/fpsyt.2025.1695846)
Supplement: Supplementary file 1 [file Supplementaryfile1.docx]

*Supplementary materials for*

Altered Language–Salience Network Connectivity in Schizophrenia and Differential Associations with Emotion Regulation

Margherita Biondi, Marco Marino, Dante Mantini and Chiara Spironelli

**Section 1. Quality control of MRI data and empirical evaluation of residual noise in the functional connectivity (FC) estimations**

We report in Table S1 the main indicators related to the correction of movement artifacts for both samples.

**Table S1.** Framewise Displacement (FD) values, percentages (%) of scrubbed volumes, and correlations between functional connectivity of each ROI and participants’ head motion for healthy controls (HC) and schizophrenia patients (SZ). No significant differences were found.

|  | **Healthy control (HC) group** | **Schizophrenia patient (SZ) group** | **Between-group Statistics** |
| --- | --- | --- | --- |
| **FD mean ± standard deviation** | 0.35 ± 0.03 | 0.46 ± 0.04 | Wilcoxon Rank Sum Test:  *z* = -1.64, *p* = 0.1 |
| **% of scrubbed volumes** | 11.3% | 24.3% | z-test: *z* = -1.499, *p* = 0.19 (*) |
| **Correlation between motion and the functional connectivity of each ROI** |  |  | z-test on Fisher z-transformed correlation coefficients: |
| **rTPJ** | 0.004 | -0.016 | *z* = 0.10, *p* = 0.92 |
| **rIFG** | 0.028 | -0.014 | *z* = 0.22, *p* = 0.83 |
| **lTPJ** | 0.004 | 0.034 | *z* = -0.15, *p* = 0.88 |
| **lIFG** | -0.005 | 0.012 | *z* = -0.09, *p* = 0.93 |
| **lAI** | -0.011 | 0.022 | *z* = -0.17, *p* = 0.86 |
| **rAI** | 0.003 | 0.020 | *z* = -0.09, *p* = 0.93 |
| **dACC** | -0.009 | -0.002 | *z* = -0.04, *p* = 0.97 |

*rTPJ = right Temporal-Parietal Junction; rIFG = right Inferior Frontal Gyrus; lTPJ = left Temporal-Parietal Junction; lIFG = left Inferior Frontal Gyrus; lAI = left Anterior Insula; rAI = right Anterior Insula; dACC = dorsal Anterio Cingulate Cortex.*

(*) Difference in percent required at a 95% confidence level is 14.33%

**Section 2. Correlations between functional connectivity of Salience Network, Language Network and Ventral Attention Network with general cognitive indices.**

To test whether the connectivity patterns considered in the main text are specific to emotion regulation or they may depend on patients’ general cognitive deficits, we correlated the connectivity values between all possible pairs of ROI for HC and SZ group with their scores of the Wechsler Abbreviated Scale of Intelligence (WASI), a tool providing an estimation of an individual’s general intellectual ability by assessing the verbal, nonverbal, and general cognition. As can be seen in the left panels of Figures S1-S3, no significant associations were found in HC adults.

Considering SZ patients, there was a significant positive association between the lIFG-lTPJ connectivity values and the WASI VERBAL IQ scores (*q* < 0.05 FDR-BY corrected), as can be seen in the right panel of Figure S2. In our SZ sample, the greater the connectivity between these two nodes, the higher their scores at the verbal subtests of the WASI.

**
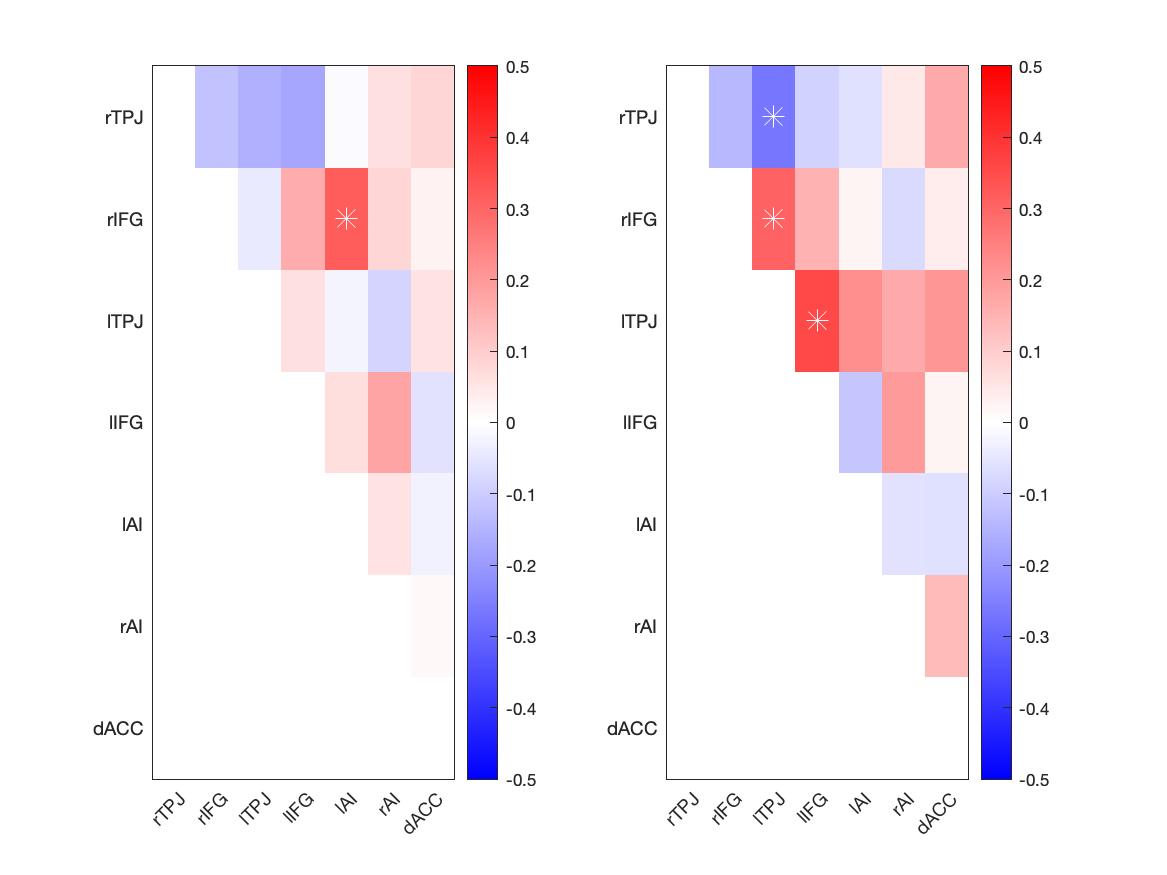
**

**Figure S1.** Correlation between WASI TOTAL IQ scores and the connectivity values between all possible pairs of ROI for HC (left panel) and SZ (right panel) group. Correlations with *p* < 0.05 are marked with an asterisk, but none survived with *q* < 0.05 (FDR-BY corrected). Red color marks positive associations, whereas blue color negative ones.


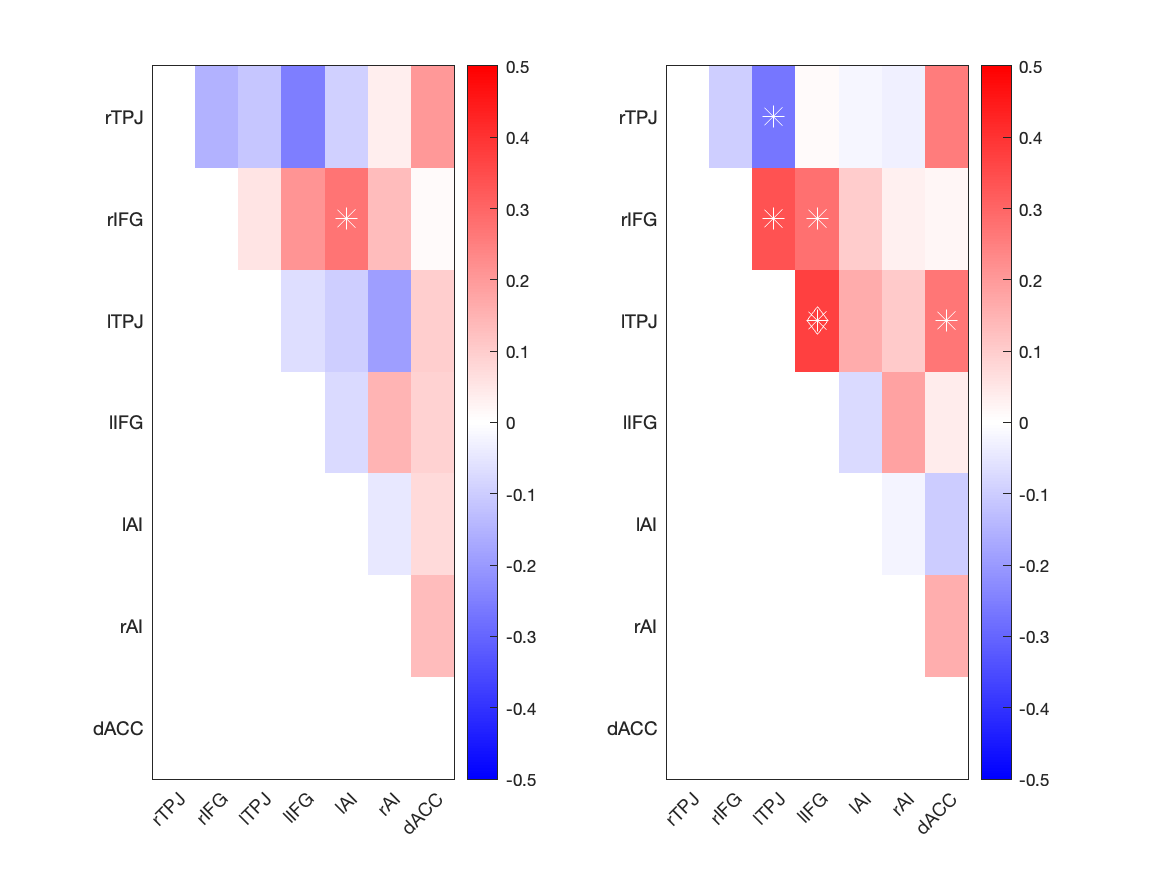


**Figure S2.** Correlation between WASI VERBAL IQ scores and the connectivity values between all possible pairs of ROI for HC (left panel) and SZ (right panel) group. Correlations with *p* < 0.05 are marked with an asterisk, that at *q* < 0.05 (FDR-BY corrected) with a diamond. Red color marks positive correlations, whereas blue color negative correlations.


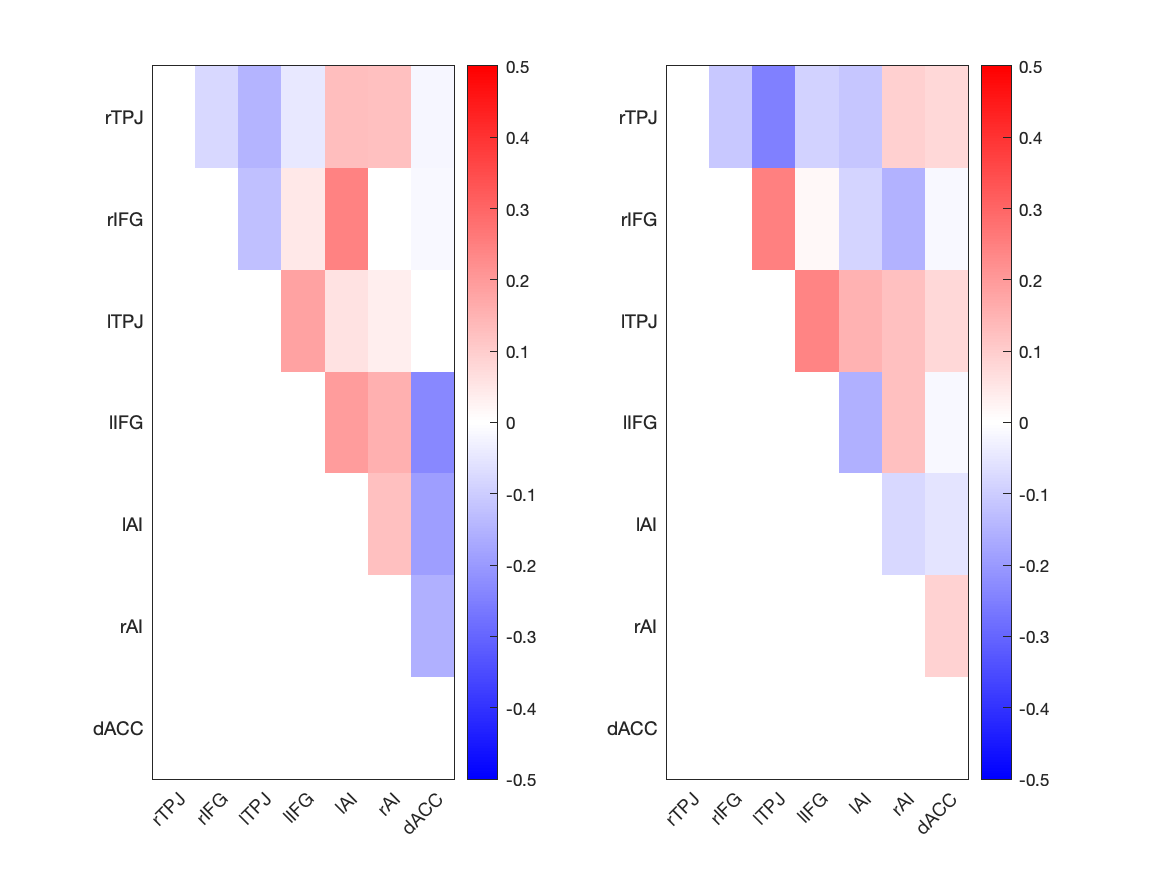


**Figure S3.** Correlation between WASI PERFORMANCE IQ scores and the connectivity values between all possible pairs of ROI for HC (left panel) and SZ (right panel) group. No significant correlations were found. Red color marks positive associations, whereas blue color negative ones.

**Section 3. Correlation between SZ patients’ functional connectivity and their pharmacological treatments**

The medication doses of our clinical sample show large variability (e.g., CPZ: 388.95 ± 307.75 mg/day): for this reason, we decided to verify the impact of Olanzapine-equivalent (OLZ-E) and Chlorpromazine-equivalent (CPZ-E) doses on both patients’ behavioral data and patients’ functional connectivity.

Behavioral analysis revealed significant correlations between drugs and patients’ behavioral performance neither on MSCEIT-ME scores (OLZ-E doses: *r* = -0.023,  *p* = 0.87; CPZ-E doses = *r* = -0.053,  *p* = 0.70) nor on WASI scales (WASI total IQ = OLZ-E doses: *r* = 0.071,  *p* = 0.60; CPZ-E doses = *r* = -0.066,  *p* = 0.63; WASI verbal IQ = OLZ-E doses: *r* = 0.099,  *p* = 0.47; CPZ-E doses = *r* = -0.010,  *p* = 0.94; WASI performance IQ = OLZ-E doses: *r* = 0.025,  *p* = 0.85; CPZ-E doses = *r* = -0.113,  *p* = 0.41).

With respect to patients’ functional connectivity, there was a significant negative association between the lIFG-rTPJ and the dosage of CPZ-equivalents, as can be seen in Figure S4. The lower the connectivity between these two nodes, the higher the dose of CPZ-equivalents in SZ patients.


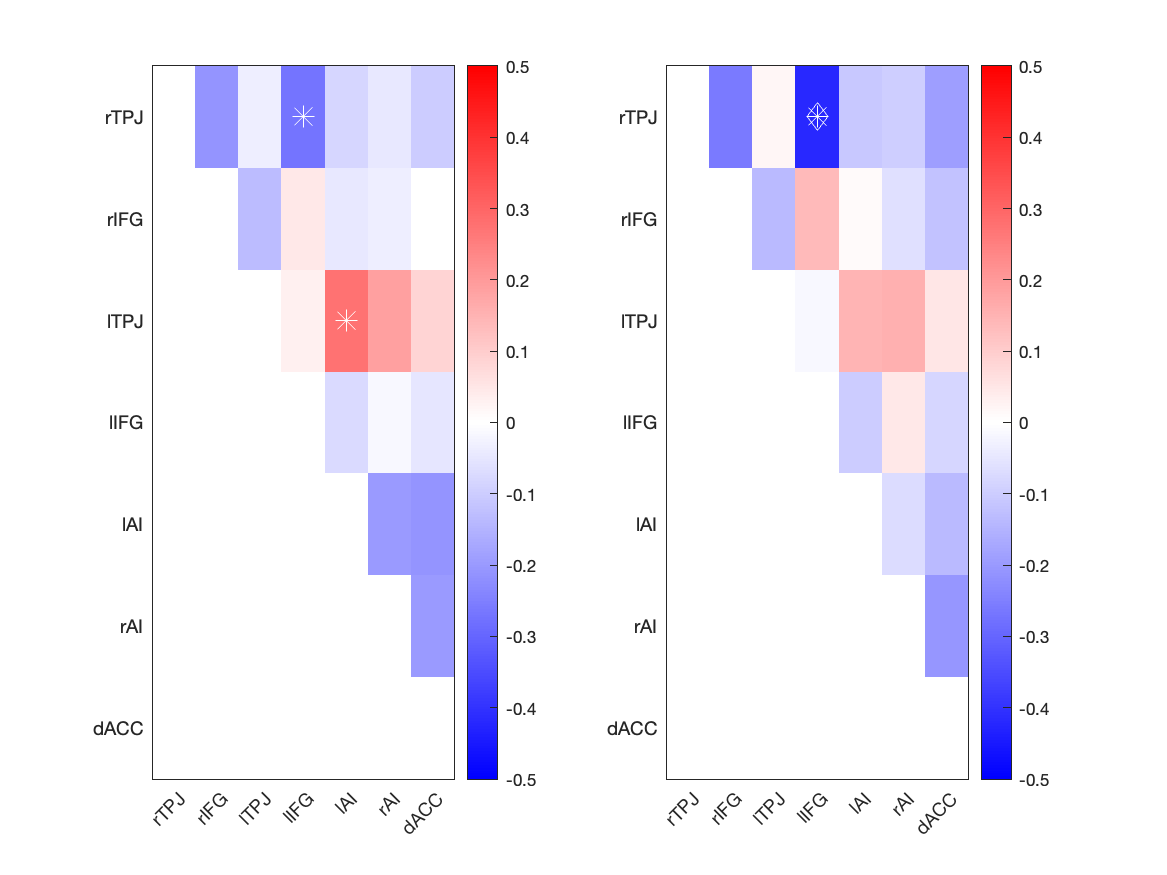


**Figure S4.** Correlation between Olanzapine-equivalent doses (left panel) and Chlorpromazine-equivalent doses (right panel) and patients’ connectivity values between all possible pairs of ROI. Correlations with *p* < 0.05 are marked with an asterisk, that at *q* < 0.05 (FDR-BY corrected) with a diamond. Red color marks positive correlations, whereas blue color negative correlations.

**Section 4. Functional connectivity analyses of other resting state networks previously implicated in emotion regulation and schizophrenia**

As past research has consistently demonstrated that emotion regulation involves the interplay between executive control/frontoparietal networks and default mode network regions, we decided to carry out a complementary analysis focused on these rs-fMRI networks. Figure S5 shows the seed position of these new set of networks, whereas seed coordinates are listed in Table S2.

**
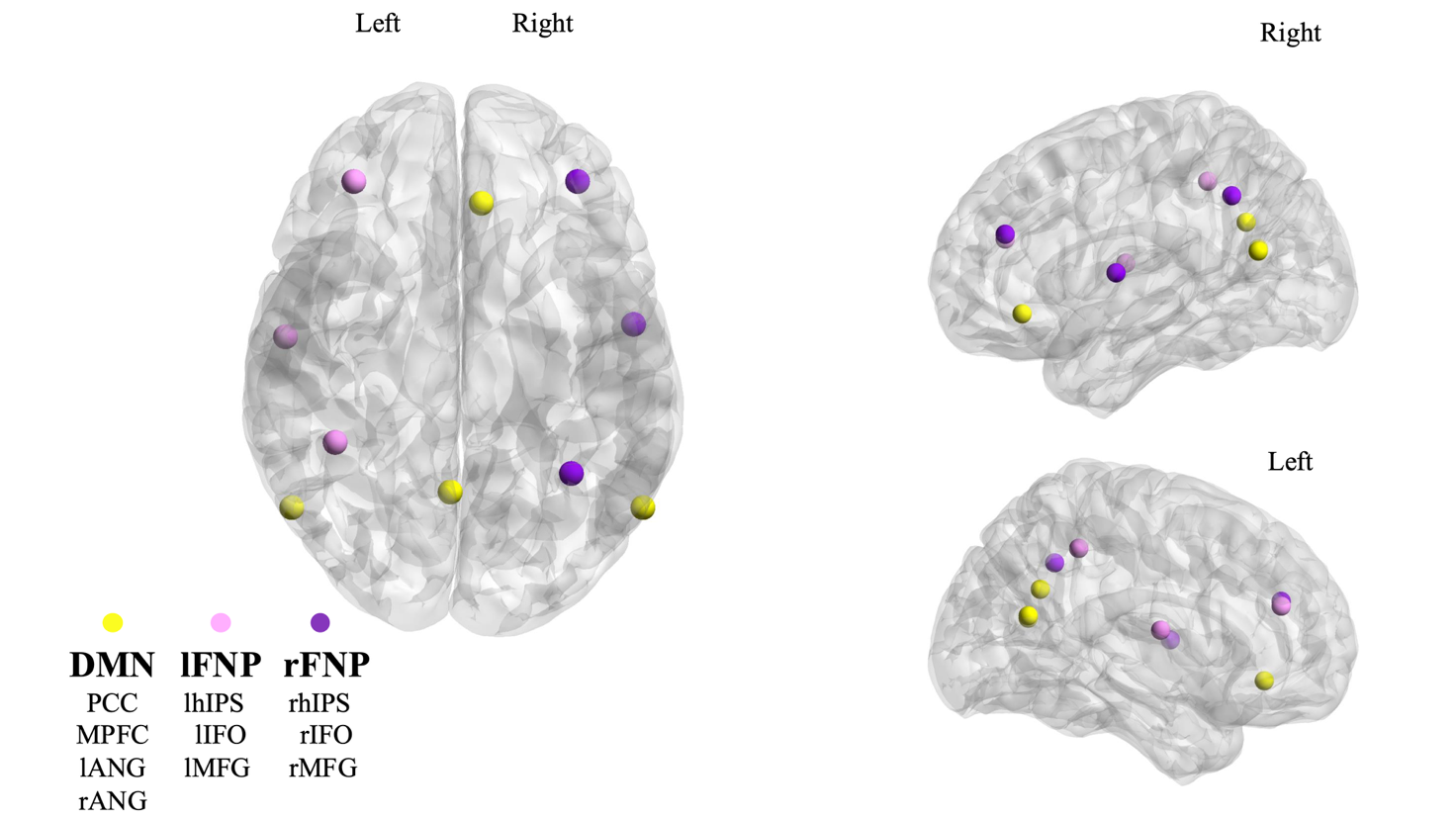
**

**Figure S5**. Anatomical positions of the 10 ROIs not considered in the main study, but significantly associated with emotional regulation in past literature, subdivided into the corresponding 3 RSNs: Default Mode Network (DMN, yellow), left Fronto-Parietal Network (lFPN, pink), right Fronto-Parietal Network (rFPN, purple). MNI coordinates of the ROIs can be found in Table S2, whereas the full names are listed here: Middle Prefrontal Cortex (MPFC), Posterior Cingulate Cortex (PCC), left Angular Gyrus (lANG), right Angular Gyrus (rANG); left Middle Frontal Gyrus (lMFG), left Inferior Frontal Operculum (lIFO), left Intra-Parietal Sulcus (lhIPS); right Middle Frontal Gyrus (rMFG), right Inferior Frontal Operculum (rIFO), right Intra-Parietal Sulcus (rhIPS).

**Table S2.** List of ROIs, with their respective MNI spatial coordinates, of the RSNs of interest.

| RSN | ROI | MNI coordinates |
| --- | --- | --- |
| **Default Mode Network (DMN)** | MPFC (Middle Prefrontal Cortex  PCC (Posterior Cingulate Cortex)  lANG (left Angular Gyrus)  rANG (right Angular Gyrus) | [-5,35,-9]  [5,-58,29]  [-57,-63,17]  [56,-63,18] |
| **Left Fronto-Parietal Network (lFPN)** | lMFG (left middle frontal gyrus)  lIFO (left inferior frontal operculum)  lhIPS (left intra-parietal sulcus) | [-36,42,24]  [-54,-4,8]  [-34,-52,40] |
| **Right Fronto-Parietal Network (rFPN)** | rMFG (right middle frontal gyrus)  rIFO (right inferior frontal operculum)  rhIPS (right intra-parietal sulcus) | [36,42,22]  [58,-8,12]  [42,-42,46] |

As can be seen in Figure S6, groups showed no significant group differences (*q* < 0.05, FDR-BY corrected) in the new ROIs.


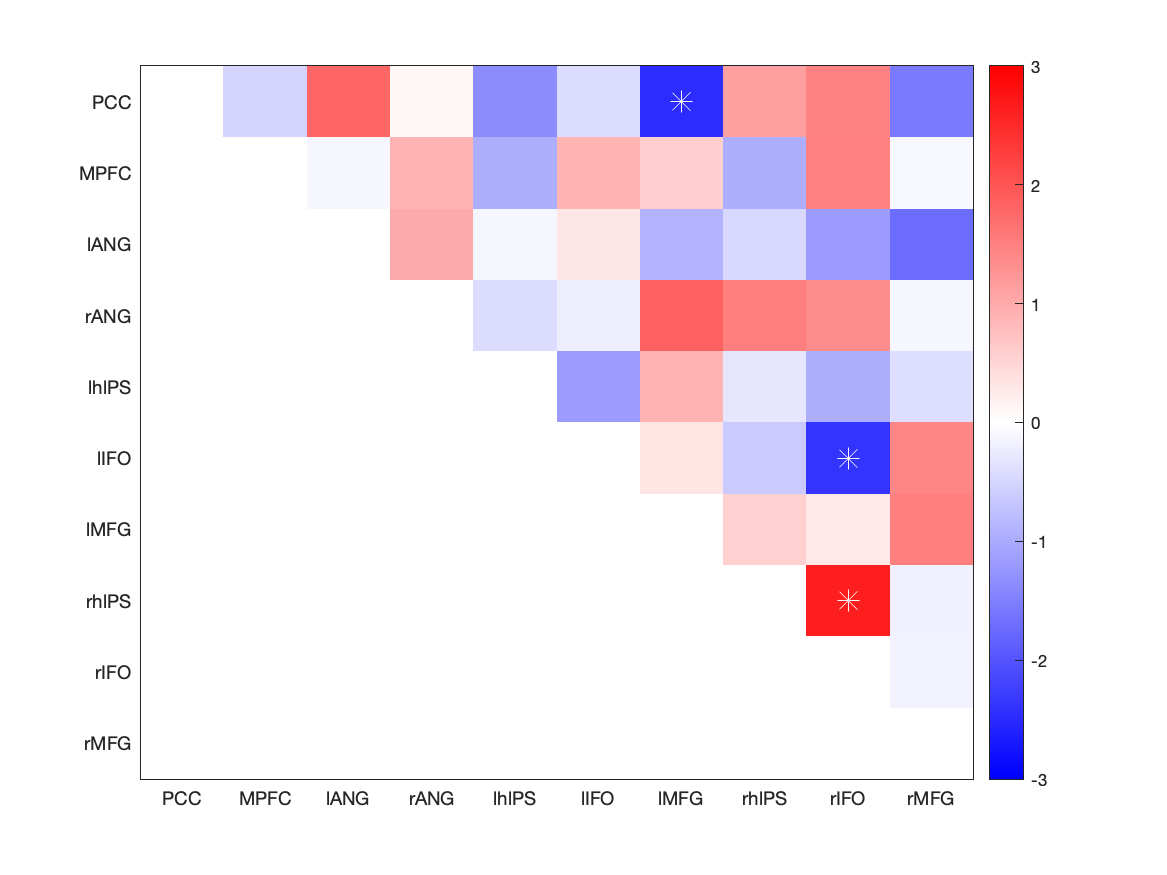


**Figure S6.** Functional connectivity values and their differences between all possible pairs of the new set of ROIs for HC and SZ groups. The matrix represents for each ROI pair significant differences between HC and SZ groups at *p* < 0.05 (asterisks). However, none of them survived FDR-BY correction (*q* < 0.05). Red color refers to HC connectivity levels greater than SZ patients, whereas blue color to greater SZ connectivity levels that HC adults.

Considering the relationship with MSCEIT-ME scores, HC showed no significant correlations with all possible pairs of the new set of ROIs (left panel of Figure S7). Conversely, SZ patients showed a negative correlation between lIFO-rhIPS connectivity and MSCEIT-ME scores: the lower the connectivity between these two nodes, the higher the patients’ scores associated with the emotional management.


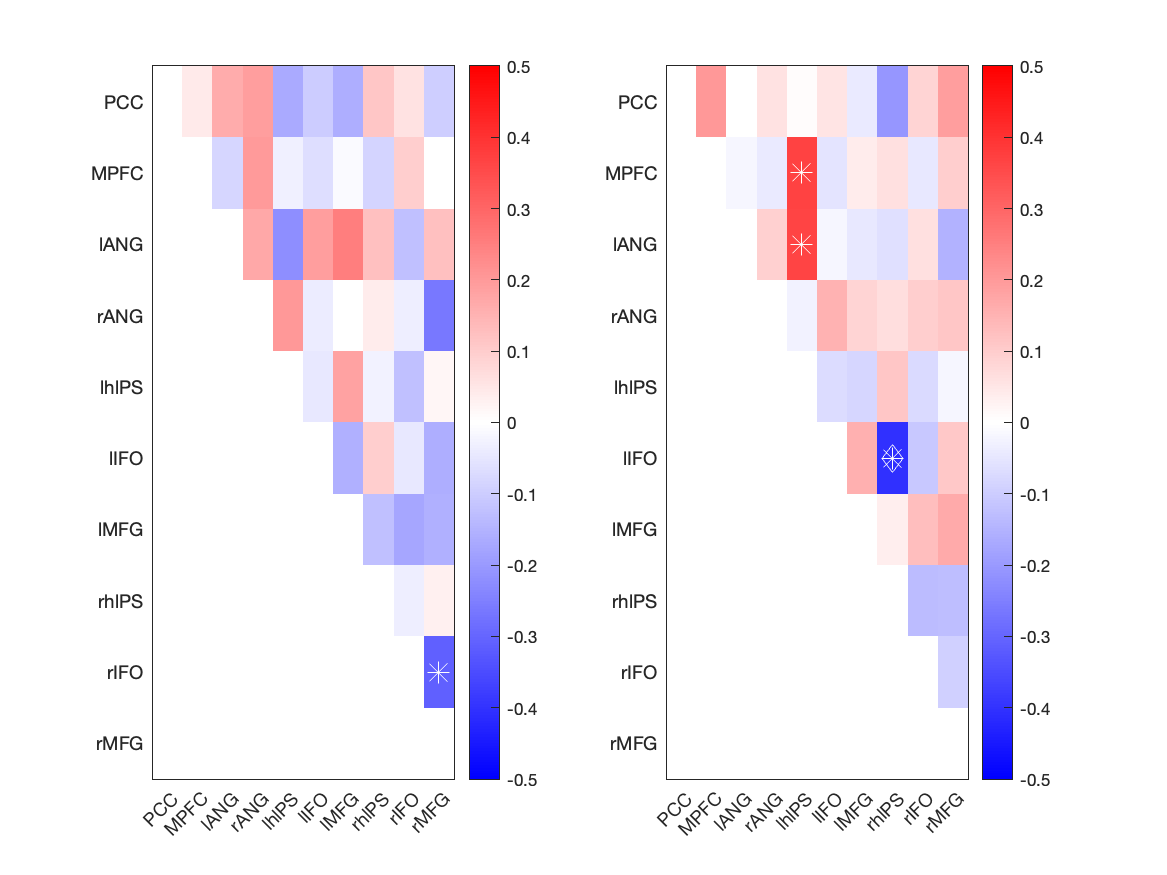


**Figure S7.** Correlations between MSCEIT-ME scores and the connectivity values between all possible pairs of the new set of ROIs for HC (left panel) and SZ (right panel) groups. Correlations with *p* < 0.05 are marked with an asterisk, that at *q* < 0.05 (FDR-BY corrected) with a diamond. Red color marks positive correlations, whereas blue color negative correlations.
